# Survival of HIV-positive patients starting antiretroviral therapy between 1996 and 2013: a collaborative analysis of cohort studies

**DOI:** 10.1016/S2352-3018(17)30066-8

**Published:** 2017-05-10

**Authors:** The Antiretroviral Therapy Cohort Collaboration, Adam Trickey, Margaret T May, Jorg-Janne Vehreschild, Niels Obel, M John Gill, Heidi M Crane, Christoph Boesecke, Sophie Patterson, Sophie Grabar, Charles Cazanave, Matthias Cavassini, Leah Shepherd, Antonella d'Arminio Monforte, Ard van Sighem, Mike Saag, Fiona Lampe, Vicky Hernando, Marta Montero, Robert Zangerle, Amy C Justice, Timothy Sterling, Suzanne M Ingle, Jonathan A C Sterne

## Abstract

**Background:**

Health care for people living with HIV has improved substantially in the past two decades. Robust estimates of how these improvements have affected prognosis and life expectancy are of utmost importance to patients, clinicians, and health-care planners. We examined changes in 3 year survival and life expectancy of patients starting combination antiretroviral therapy (ART) between 1996 and 2013.

**Methods:**

We analysed data from 18 European and North American HIV-1 cohorts. Patients (aged ≥16 years) were eligible for this analysis if they had started ART with three or more drugs between 1996 and 2010 and had at least 3 years of potential follow-up. We estimated adjusted (for age, sex, AIDS, risk group, CD4 cell count, and HIV-1 RNA at start of ART) all-cause and cause-specific mortality hazard ratios (HRs) for the first year after ART initiation and the second and third years after ART initiation in four calendar periods (1996–99, 2000–03 [comparator], 2004–07, 2008–10). We estimated life expectancy by calendar period of initiation of ART.

**Findings:**

88 504 patients were included in our analyses, of whom 2106 died during the first year of ART and 2302 died during the second or third year of ART. Patients starting ART in 2008–10 had lower all-cause mortality in the first year after ART initiation than did patients starting ART in 2000–03 (adjusted HR 0·71, 95% CI 0·61–0·83). All-cause mortality in the second and third years after initiation of ART was also lower in patients who started ART in 2008–10 than in those who started in 2000–03 (0·57, 0·49–0·67); this decrease was not fully explained by viral load and CD4 cell count at 1 year. Rates of non-AIDS deaths were lower in patients who started ART in 2008–10 (*vs* 2000–03) in the first year (0·48, 0·34–0·67) and second and third years (0·29, 0·21–0·40) after initiation of ART. Between 1996 and 2010, life expectancy in 20-year-old patients starting ART increased by about 9 years in women and 10 years in men.

**Interpretation:**

Even in the late ART era, survival during the first 3 years of ART continues to improve, which probably reflects transition to less toxic antiretroviral drugs, improved adherence, prophylactic measures, and management of comorbidity. Prognostic models and life expectancy estimates should be updated to account for these improvements.

**Funding:**

UK Medical Research Council, UK Department for International Development, EU EDCTP2 programme.

## Introduction

For 20 years, combination antiretroviral therapy (ART) has been the standard approach to treating HIV-1 infection in Europe and North America. The first ART regimens were inferior to those currently available, which better suppress HIV replication, are less toxic, and have higher genetic barriers to resistance, reduced pill burden (often one a day), and fewer side-effects.^1^, ^2^ Other improvements in health care since 1996 for people living with HIV include treatment and prophylaxis for opportunistic infections and management of comorbidities.^3^ Improvements in intensive care management, disease screening, and health promotion might also have improved prognosis. Therefore, people living with HIV who started ART more recently might have improved survival compared with those treated earlier in the ART era.

The Antiretroviral Therapy Cohort Collaboration (ART-CC) previously reported that despite improvements in virological response to ART, mortality 1 year after initiation of ART did not decrease between 1998 and 2003.^4^ This absence of improvement in survival might have been related to changes in patients' characteristics: increasing numbers were from areas with a high prevalence of tuberculosis infection.^4^ Some studies have reported improvements in overall survival and changing causes of death, with proportionately fewer AIDS-related deaths in more recent years,^5^, ^6^, ^7^, ^8^, ^9^ but none has investigated trends in prognosis after starting ART by calendar period. We examined changes in all-cause and cause-specific mortality in the first 3 years of ART during 1996–2013, and investigated trends in life expectancy.

## Methods

### Participants

We combined data from 18 European and North American HIV cohorts participating in ART-CC, which includes ART-naive people living with HIV aged 16 years or older who started treatment with three or more antiretroviral drugs between 1996 and 2010.^10^ Cohorts were approved by ethics committees or institutional review boards, used standardised data collection methods, and scheduled follow-up visits at least every 6 months. Cohorts included in this paper were the French Hospital Database on HIV (FHDH); the Italian Cohort of Antiretroviral-naive patients (ICONA); the Swiss HIV Cohort Study (SHCS); the AIDS Therapy Evaluation project, Netherlands (ATHENA); the Multicenter Study Group on EuroSIDA; the Aquitaine Cohort, France; the Royal Free Hospital Cohort, UK; the South Alberta Clinic Cohort, Canada; the Danish HIV Cohort Study, Denmark; HAART Observational Medical Evaluation and Research (HOMER) Cohort, Canada; HIV Atlanta Veterans Affairs Cohort Study (HAVACS), USA; Osterreichische HIV-Kohortenstudie (OEHIVKOS), Austria; Proyecto para la Informatizacion del Seguimiento Clinico-epidemiologico de la Infeccion por HIV y SIDA (PISCIS), Spain; University of Washington HIV Cohort, USA; VACH, Spain; Veterans Aging Cohort Study (VACS), USA; Vanderbilt, USA; and the Koln/Bonn Cohort, Germany.

**Research in context**

**Evidence before this study**

We ran three PubMed searches for articles published between Jan 1, 2001, and June 1, 2016, with the terms (1) “HIV”, “calendar year”, and “mortality”; (2) “HIV”, “life expectancy”, and “mortality”; (3) “HIV”, “causes of death”, and “mortality”. A study of 2675 patients in the Australian HIV cohort found lower mortality in individuals who started antiretroviral therapy (ART) from 2004 onwards compared with earlier years, although that study focused on the effect of duration of treatment rather than trends in early mortality on ART. A large study by D:A:D found lower all-cause, cardiovascular, and liver disease mortality in patients followed up in 2009–11 compared with 1999–2000, but did not analyse deaths by period of starting ART. We previously reported that between 1995 and 2003, virological response to ART improved but early mortality did not decrease. Surveillance data from the USA showed increasing life expectancy with year of HIV diagnosis between 1996 and 2005. We also reported that life expectancy in individuals starting ART increased between 1996 and 2005; studies in Europe and the USA have also shown increases in life expectancy over time.

**Added value of this study**

Our study, based on a large collaboration of cohorts in Europe and North America, found that substantial declines in mortality for individuals starting ART in 2008–10, compared with earlier years, has resulted in increased life expectancy. However, this life expectancy remains lower than that of the general population. Declines in mortality were greater for the second and third years after starting ART than for the first year after starting ART. Rates of non-AIDS-related deaths, particularly deaths from cardiovascular disease, were substantially lower in 2008–10 than previously. There was little evidence that mortality has declined in people who inject drugs.

**Implications of all the available evidence**

Improvements in the care of people living with HIV since the introduction of ART 20 years ago have led to improved survival and increased life expectancy in those starting ART. These improvements probably reflect the availability of superior antiretroviral agents, more options for the management of patients developing resistance, fewer drug interactions, better management of opportunistic infections and chronic diseases, and introduction of screening and prevention programmes. Prognostic models and estimates of life expectancy should be updated to account for these improvements.

Eligible patients started ART at least 3 years before the cohort-specific database close date, which varied from May 31, 2012, to July 31, 2013, and had a baseline CD4 cell count measured within a window 3 months before until 2 weeks after starting ART. We defined CD4 cell count and viral load 1 year after the start of ART as the closest measurement before 1 year within a 9–12 month window.

Patients were followed up for all-cause and cause-specific mortality from the time of starting ART, considered lost to follow-up if there was a gap of more than 1 year between the dates they were last known to be alive and the database close date, and censored 6 months after the last recorded measurement. Mortality information was obtained through linkage with Vital Statistics agencies and hospitals or physician report and active follow-up of participants. Methods for classifying causes of death, with an adaptation of the CoDe project protocol, are described elsewhere.^11^ Deaths were coded as AIDS-related if there was a serious AIDS defining condition close to death or a low CD4 cell count (<100 cells per μL) before death, and a diagnosis compatible with AIDS as cause of death.

### Statistical analysis

We compared characteristics of patients by calendar period of initiation of ART (1996–99, 2000–03, 2004–07, 2008–10). We used Cox models stratified by cohort to estimate unadjusted and adjusted mortality hazard ratios (HRs) by period of initiation of ART. Models were adjusted for sex, injecting drug use, AIDS at baseline, age (16–29, 30–39, 40–49, 50–59, ≥60 years), CD4 cell count (0–24, 25–49, 50–99, 100–199, 200–349, 350–499, ≥500 cells per μL), and HIV-1 RNA viral load (0 to <10 000, ≥10 000 to <100 000, ≥100 000 copies per mL) at the start of ART. Because mortality is higher in the first year of ART, we fitted separate models for the first year after starting ART and the second and third years after starting ART. To investigate the mediating effect of response to therapy, we additionally adjusted the second and third year analysis for CD4 cell count and viral load measured 1 year after initiation of ART. We compared HR for regimen failure, defined as switching regimen within 6 months of a viral load measurement of more than 1000 copies per mL after first achieving viral suppression, by period of ART initiation. In patients with regimen failure, we estimated mortality HR by period of ART initiation.

We used Cox models to investigate the consistency of mortality trends across subgroups of patients defined by sex and transmission risk (men who have sex with men [MSM], male heterosexual, female heterosexual, men who inject drugs, women who inject drugs) in European patients (because transmission risk was missing for large numbers of North American patients); CD4 cell count categories (<100, 100–199, 200–349, ≥350 cells per μL); age; and region (Europe, North America). In a sensitivity analysis, patients were considered lost to follow-up at 6 months rather than 12 months. We repeated analyses with the following causes of death as the outcome: AIDS-related, non-AIDS-related (ie, non-AIDS infection, malignancies not caused by AIDS or hepatitis, liver disease, cardiovascular disease, and other [causes with ≤20 cases]), and unnatural causes (suicide, accident or other violent death, euthanasia, and substance abuse), and those missing or unknown. We estimated sex-specific life expectancy by period of ART initiation, overall and by region (North America, Europe). We used a Poisson model to estimate mortality in age bands of 5 years, which were used to construct life tables and estimate average age at death for those aged 20 years at initiation of ART. We compared these estimates with those from the French and US general populations. For comparability across periods and to investigate the effect of higher mortality in the first year of ART, we estimated life expectancy on the basis of mortality in the first 3 years of follow-up, and then with mortality in the second and third years of follow-up. Because there were few patients aged 70 years or older, for the oldest open-ended age group we used the French general population mortality multiplied by the mean rate ratio in ART-CC compared with the French general population (chosen because France contributed the most patients) for ages 60–64 and 65–69 years. Per-period changes in life expectancy were estimated with metaregression. We used Stata (version 14) for analyses.

### Role of the funding source

The funders of the study, the UK Medical Research Council, UK Department for International Development, and the European Union, had no role in study design, data collection, data analysis, data interpretation, or writing of the report. The corresponding author had full access to all the data in the study and had final responsibility for the decision to submit for publication.

## Results

88 504 patients were eligible for our analyses. During 84 621 person-years, 2106 (2%) patients died in the first year after starting ART (24·9 per 1000 person-years). 81 608 (92%) individuals remained in the study for more than 1 year, of whom 2302 (3%) died during 153 813 person-years (15·0 per 1000 person-years). 4594 (5%) patients were lost to follow-up during the first year after starting ART and 6674 patients (8%) were lost-to follow-up during the second and third years. Patients who were lost to follow-up had lower viral loads and higher CD4 cell counts than were those not lost to follow-up, and they were also more likely to be female, younger, and people who inject drugs (data not shown).

The proportion of women increased from 20% in 1996–99 to 28% in 2004–07, then decreased to 21% in 2008–10 (table 1). Median age increased between 1996–99 and 2008–10, whereas the proportion of people who inject drugs decreased from 17% to 7%. Median CD4 count 1 year after ART initiation increased substantially, from 370 cells per μL (IQR 211–572) in 1996–99 to 430 cells per μL (295–570) in 2008–10, and the proportion of patients with HIV-1 RNA viral load of 500 copies per mL or lower increased from 71% in 1996–99 to 93% in 2008–10.

**Table 1:** Characteristics of patients at the time of starting antiretroviral therapy with number of deaths, by period of initiation

		**1996–99**	**2000–03**	**2004–07**	**2008–10**
Total deaths		1431/24 445 (6%)	1452/25 683 (6%)	1084/24 462 (4%)	441/13 914 (3%)
Men		1240/19 529 (6%)	1231/18 653 (7%)	882/17 570 (5%)	377/10 995 (3%)
Women		191/4916 (4%)	221/7030 (3%)	202/6892 (3%)	64/2919 (2%)
No injecting drug use		1161/20 186 (6%)	1187/22 182 (5%)	877/22 020 (4%)	385/12 885 (3%)
Injecting drug use		270/4259 (6%)	265/3501 (8%)	207/2442 (8%)	56/1029 (5%)
No AIDS		810/19 118 (4%)	799/19 589 (4%)	595/19 262 (3%)	239/11 570 (2%)
AIDS		621/5327 (12%)	653/6094 (11%)	489/5200 (9%)	202/2344 (9%)
Age (years)		36 (31–43)	37 (31–45)	39 (32–46)	40 (32–47)
	16–29	117/4457 (3%)	95/4620 (2%)	55/4118 (1%)	23/2208 (1%)
	30–39	508/11 176 (5%)	399/10 320 (4%)	256/8846 (3%)	74/4651 (2%)
	40–49	433/5729 (8%)	485/6714 (7%)	352/7294 (5%)	142/4311 (3%)
	50–59	257/2270 (11%)	313/2998 (10%)	261/3003 (9%)	115/1919 (6%)
	≥60	116/813 (14%)	160/1031 (16%)	160/1201 (13%)	87/825 (11%)
CD4 count (cells per μL)		238 (93–394)	200 (81–326)	219 (115–310)	265 (157–351)
	0–24	356/2511 (14%)	355/2789 (13%)	232/1921 (12%)	97/849 (11%)
	25–49	173/1508 (11%)	179/1753 (10%)	102/1291 (8%)	37/559 (7%)
	50–99	197/2332 (8%)	244/2771 (9%)	173/2203 (8%)	57/891 (6%)
	100–199	288/4212 (7%)	331/5461 (6%)	246/5370 (5%)	69/2272 (3%)
	200–349	237/6171 (4%)	217/7354 (3%)	240/9090 (3%)	115/5764 (2%)
	350–499	109/4258 (3%)	76/3171 (2%)	58/2673 (2%)	44/2384 (2%)
	≥500	71/3453 (2%)	50/2384 (2%)	33/1914 (2%)	22/1195 (2%)
HIV-1 RNA (log copies per mL)		4·9 (4·2–5·4)	4·9 (4·3–5·4)	4·8 (4·1–5·3)	4·7 (4·1–5·2)
	0–3·99	177/4439 (4%)	179/4693 (4%)	132/5317 (2%)	70/3224 (2%)
	4–4·99	396/9397 (4%)	426/9355 (5%)	332/8937 (4%)	158/5675 (3%)
	≥5	858/10 609 (8%)	847/11 635 (7%)	620/10 208 (6%)	213/5015 (4%)
Regimen					
	NNRTI-based	210/4178 (5%)	609/11 391 (5%)	460/12 126 (4%)	215/7902 (3%)
	Protease inhibitor-based	1159/19 184 (6%)	602/9520 (6%)	555/10 496 (5%)	197/5398 (4%)
	NRTI/abacavir*	18/364 (5%)	196/4040 (5%)	47/1312 (4%)	4/117 (3%)
	NRTI/non-abacavir†	28/542 (5%)	29/564 (5%)	6/240 (3%)	0/29 (0%)
	Other	16/177 (9%)	16/168 (10%)	16/288 (6%)	25/468 (5%)

Data are number of deaths/number of patients (%) or median (IQR). NNRTI=non-nucleoside reverse transcriptase inhibitor. NRTI=nucleoside reverse transcriptase inhibitor.

* Triple NRTI including abacavir.

† Triple NRTI not including abacavir.

During 1996–99, most patients started a protease inhibitor-based regimen, whereas after 2000 non-nucleoside reverse transcriptase inhibitor (NNRTI)-based regimens were most common (table 2). The protease inhibitors indinavir, nelfinavir, and saquinavir were replaced by atazanavir, darunavir, and lopinavir. Of the NNRTIs, efavirenz was the most commonly used third regimen drug from 2000 onwards. The NRTIs didanosine, stavudine, and zidovudine were replaced by abacavir and tenofovir.

**Table 2:** Proportion of patients prescribed specific antiretroviral drugs as their first regimen, by period of initiation

	**1996–99 (n=24 445)**	**2000–03 (n=25 683)**	**2004–07 (n=24 462)**	**2008–10 (n=13 914)**
**Protease inhibitors**				
Amprenavir	0	1%	0	0
Atazanavir	0	0	13%	19%
Darunavir	0	0	0	3%
Fosamprenavir	0	0	6%	2%
Indinavir	37%	9%	1%	0
Lopinavir	0	8%	16%	13%
Nelfinavir	25%	16%	3%	0
Ritonavir	7%	1%	1%	2%
Saquinavir	10%	3%	3%	1%
**Non-nucleoside reverse transcriptase inhibitors**				
Efavirenz	6%	30%	42%	50%
Nevirapine	12%	15%	8%	7%
**Entry inhibitors**				
Enfuvirtide	0	0	1%	0
**Integrase inhibitors**				
Raltegravir	0	0	0	3%
**Nucleoside reverse transcriptase inhibitors**				
Abacavir	2%	18%	16%	11%
Didanosine	17%	14%	7%	1%
Emtricitabine	0	0	37%	80%
Lamivudine	80%	90%	59%	19%
Stavudine	40%	20%	2%	0
Tenofovir	0	5%	49%	79%
Zalcitabine	3%	0	0	0
Zidovudine	59%	68%	34%	8%

**Figure 1:**
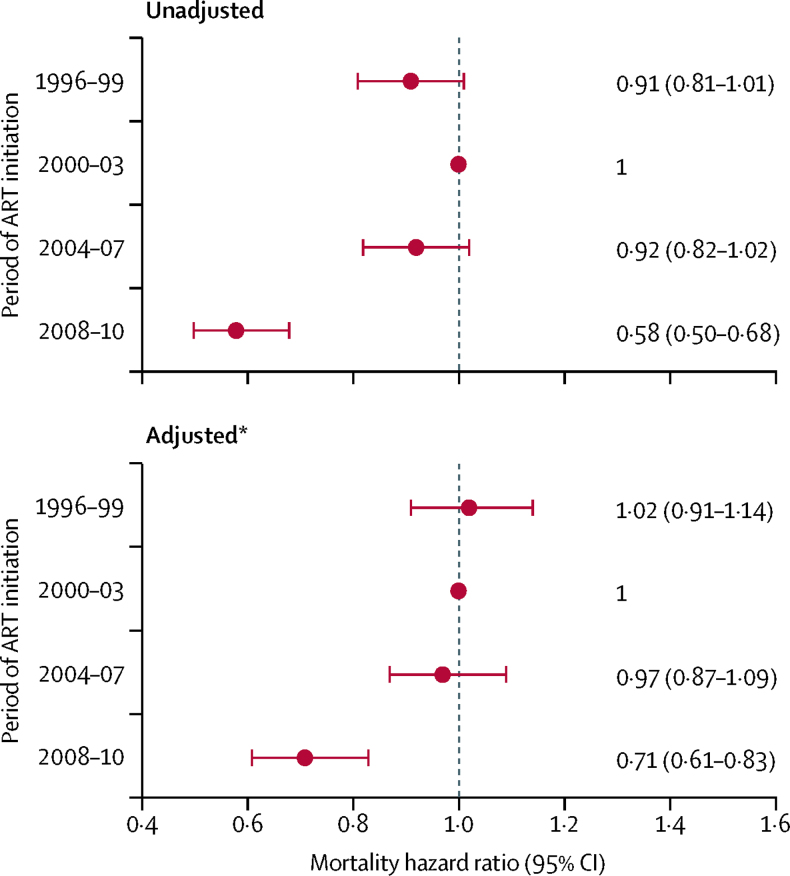
Unadjusted and adjusted all-cause mortality hazard ratios for the first year after starting antiretroviral therapy (ART), by period of initiation *Adjusted for age, sex, AIDS, risk group, CD4 cell count, and HIV-1 RNA at the time of starting ART.

Compared with patients who started ART in 2000–03, all-cause mortality during the first year of ART was similar in patients who started ART between 1996 and 2007, but substantially lower for those who started ART in 2008–10 (adjusted HR 0·71, 95% CI 0·61–0·83; figure 1). The adjusted HR per calendar period was 0·90 (0·87–0·95). Declines in 1 year mortality over calendar time were consistent across subgroups of patients defined by their characteristics at the start of ART, apart from individuals who inject drugs and those starting ART with CD4 counts less than 100 cells per μL or CD4 count greater than or equal to 350 cells per μL (appendix).

**Figure 2:**
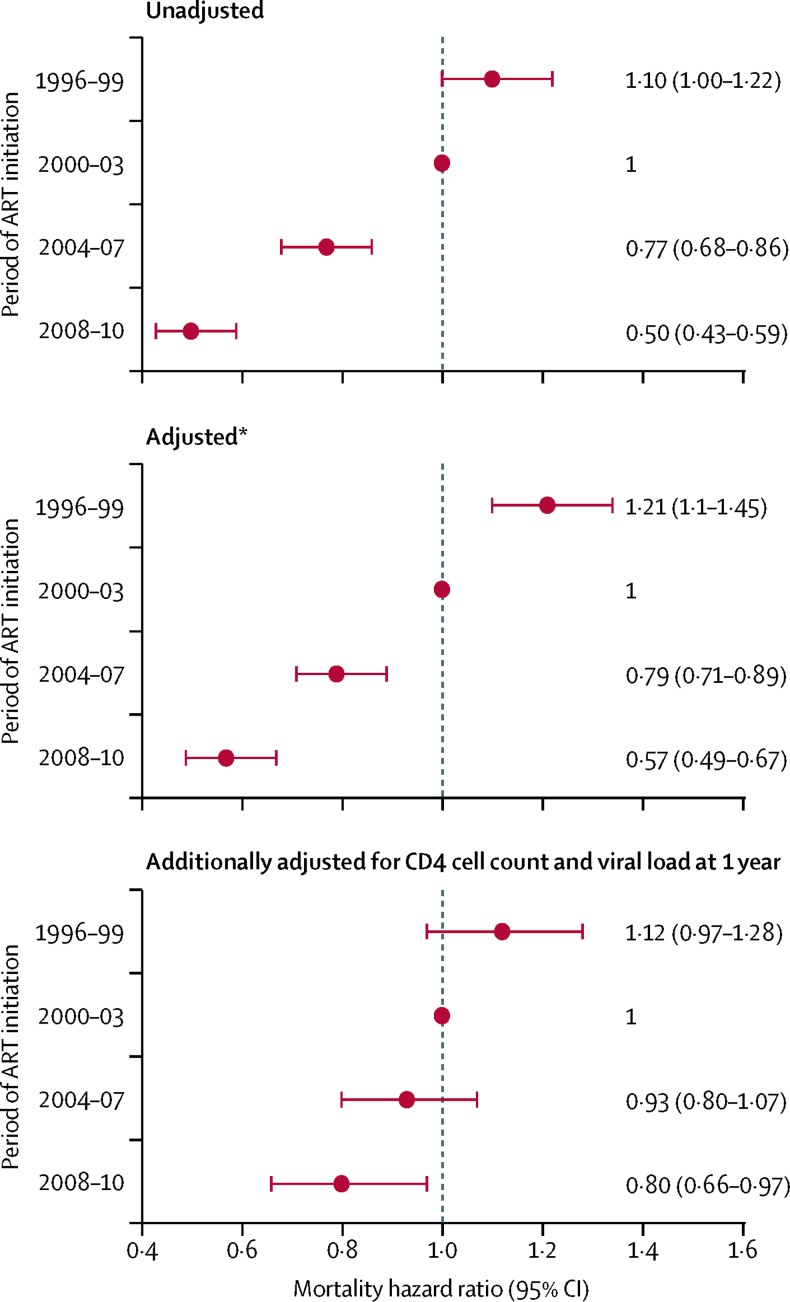
All-cause mortality hazard ratios for the second and third years after starting antiretroviral therapy (ART), by period of initiation *Adjusted for age, sex, AIDS, risk group, CD4 cell count, and HIV-1 RNA at the time of starting ART.

All-cause mortality in the second and third years after starting ART declined substantially over calendar time (adjusted HR per calendar period 0·78, 95% CI 0·75–0·82; figure 2). Declines were consistent across Europe and North America, age groups, and CD4 cell count at ART initiation. The decline in mortality was less in people who inject drugs (adjusted HR per calendar period 0·90 [0·80–1·02] for men and 0·95 [0·76–1·20] for women; appendix p 1) than in other groups. We examined the mediating effects of CD4 cell count and viral load measured 1 year after starting ART on mortality trends during the second and third years of ART in 53 244 (65%) eligible patients with available measurements (figure 2). Additionally adjusting for 1 year CD4 cell count and viral load attenuated the adjusted HR per calendar period to 0·90 (0·85–0·96). The proportion of patients with regimen failure declined over time (adjusted HR per calendar period 0·73, 0·72–0·75), but among those with regimen failure there was no evidence of an improvement in survival (adjusted HR per calendar period 0·98, 0·87–1·09; appendix p 2). Results of sensitivity analyses in which patients were considered lost to follow-up at 6 months rather than 12 months were similar to the main analyses (data not shown).

Causes were classified for 3126 (71%) of 4408 deaths. Rates of deaths from AIDS during the first year of ART declined over calendar time (adjusted HR per calendar period 0·93, 95% CI 0·86–0·99), with greater declines for the second and third year of ART (0·69, 0·64–0·76). Rates of non-AIDS-related death during the first year of follow-up declined over calendar time (0·87, 0·80–0·95), as did rates during the second and third years of follow-up (0·75, 0·69–0·81). Declines in mortality were consistent across causes of mortality (table 3): the greatest decline was in liver-related deaths during the second and third years of ART.

**Table 3:** Adjusted hazard ratios for specific causes of death by period of antiretroviral therapy (ART) initiation for first year of ART and second and third years of ART

		**Number of deaths**	**Period of ART initiation**				**Per period**
			1996–99	2000–03	2004–07	2008–10	
**First year of follow-up**							
AIDS		902	0·98 (0·83–1·16)	1	0·94 (0·79–1·11)	0·71 (0·56–0·90)	0·93 (0·86–0·99)
Non-AIDs		525	1·04 (0·83–1·30)	1	1·09 (0·88–1·35)	0·48 (0·34–0·67)	0·87 (0·80–0·95)
	Non-AIDS infection	117	0·89 (0·55–1·43)	1	1·11 (0·71–1·74)	0·45 (0·22–0·94)	0·91 (0·76–1·10)
	Non-AIDS, non-hepatitis malignancies	122	1·01 (0·61–1·68)	1	1·53 (0·97–2·39)	0·65 (0·34–1·24)	0·99 (0·83–1·18)
	Liver-related	76	1·07 (0·60–1·88)	1	0·93 (0·52–1·67)	0·36 (0·14–0·96)	0·80 (0·63–1·01)
	Cardiovascular	64	0·95 (0·52–1·73)	1	0·72 (0·39–1·35)	0·19 (0·06–0·62)	0·71 (0·55–0·92)
	Other	146	1·18 (0·78–1·78)	1	1·03 (0·67–1·59)	0·64 (0·34–1·19)	0·87 (0·73–1·02)
Unnatural*		107	1·37 (0·82–2·29)	1	1·49 (0·90–2·48)	0·62 (0·29–1·34)	0·89 (0·73–1·08)
Missing/unknown		572	1·00 (0·81–1·24)	1	0·86 (0·69–1·08)	0·98 (0·76–1·23)	0·97 (0·89–1·05)
**Second and third years of follow-up**							
AIDS		646	1·34 (1·12–1·60)	1	0·74 (0·59–0·92)	0·35 (0·24–0·51)	0·69 (0·64–0·76)
Non-AIDS		770	1·12 (0·94–1·34)	1	0·86 (0·71–1·03)	0·29 (0·21–0·40)	0·75 (0·69–0·81)
	Non-AIDS infection	132	0·79 (0·52–1·19)	1	0·66 (0·43–1·04)	0·27 (0·12–0·59)	0·79 (0·66–0·95)
	Non-AIDS, non-hepatitis malignancies	206	1·48 (1·03–2·13)	1	1·40 (0·97–2·00)	0·50 (0·28–0·87)	0·82 (0·71–0·94)
	Liver-related	127	0·94 (0·63–1·40)	1	0·49 (0·30–0·79)	0·15 (0·05–0·42)	0·66 (0·54–0·80)
	Cardiovascular	100	0·82 (0·50–1·34)	1	0·79 (0·49–1·29)	0·21 (0·08–0·53)	0·78 (0·64–0·95)
	Other	205	1·47 (1·05–2·05)	1	0·93 (0·64–1·34)	0·29 (0·14–0·59)	0·69 (0·60–0·80)
Unnatural*		176	1·06 (0·74–1·53)	1	0·91 (0·62–1·35)	0·32 (0·16–0·63)	0·79 (0·68–0·92)
Missing/unknown		710	1·23 (1·02–1·48)	1	0·73 (0·58–0·91)	1·19 (0·96–1·49)	0·93 (0·87–1·00)

Data are hazard ratio (95% CI), mutually adjusted for age, sex, AIDS, risk group, CD4 cell count, HIV-1 RNA, and stratified by cohort, with 2000–03 as comparator.

* Unnatural deaths include suicide, accident or other violent death, euthanasia, and substance abuse.

**Figure 3:**
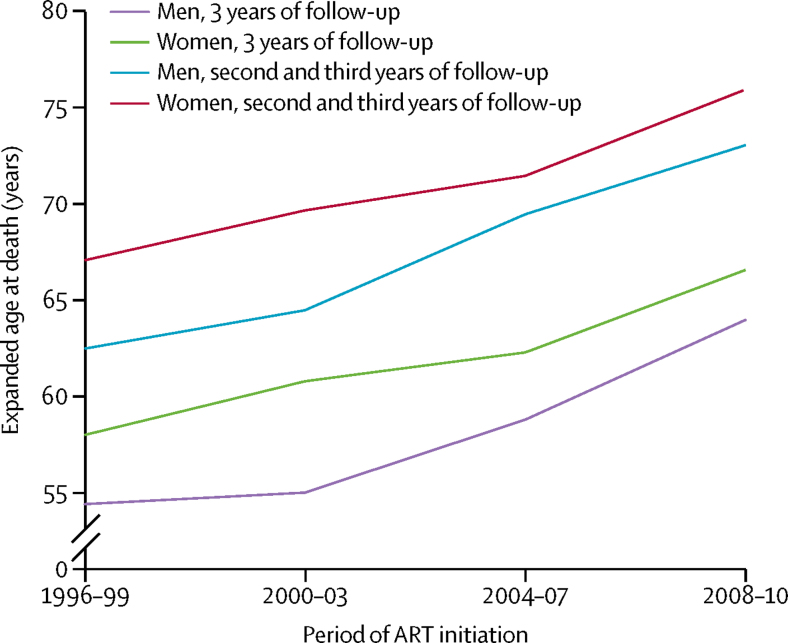
Expected age at death of men and women living with HIV starting antiretroviral therapy (ART) aged 20 years, by period of initiation Estimates of life expectancy were based on mortality during the first 3 years of follow-up and the second and third years of follow-up. Data are for all regions.

Life expectancy increased with calendar period of initiation of ART, for both men and women (figure 3 and appendix p 2). Expected average ages at death for Europeans aged 20 years starting ART in 2008–10, on the basis of mortality during the first 3 years of ART, were 67·6 years (95% CI 66·7–68·5) for men and 67·9 years (67·2–68·7) for women, lower than in the French general population (79 years in men and 85 years in women). Life expectancy was lower in North America (expected age at death 65·9 years [65·0–66·8] in men and 63·2 years [62·2–64·3] in women for patients aged 20 years starting ART in 2008–10) than in Europe and was lower than in the US general population (78 years in men and 82 years in women). When estimates of life expectancy were based on mortality during the second and third years of ART, the average ages at death were around 10 years higher. Increases in life expectancy over calendar time were similar when estimated with data for the first 3 years and for the second and third years of follow-up. The expected age at death of a 20-year-old patient starting ART during 2008–10, who had a CD4 count of more than 350 cells per μL 1 year after starting ART, was 78·0 years (77·7–78·3).

## Discussion

Between 1996 and 2013, survival of people living with HIV in the first 3 years since ART initiation improved substantially. During the first year of ART, mortality was similar in patients who started ART between 1996 and 2007, but lower during 2008–10. Survival over calendar time improved consistently during the second and third years after initiation of ART. Declines in mortality were lower in people who inject drugs than in other groups. Response to ART, measured by CD4 cell count and viral load 1 year after starting ART, only partly explained improvements in survival during the second and third years of ART. AIDS-related and non-AIDS-related mortality declined over calendar time, during the first year and second–third years after ART initiation. Life expectancy in patients starting ART has increased by about 10 years during the ART era, but remains lower than in the general population. Patients who started ART during 2008–10 whose CD4 counts exceeded 350 cells per μL 1 year after ART initiation have estimated life expectancy approaching that of the general population.

Reduced mortality during the first year of ART is likely to be explained by better initial regimens with greater effectiveness and improved tolerability with fewer side-effects, because rates of regimen modification are highest soon after starting ART.^12^ Improvements in survival during the second and third years of ART are probably caused by increased viral suppression, declining rates of viral failure, and increasing treatment options.^13^, ^14^ Simpler regimens might have contributed to improvements in both short-term and long-term adherence to ART. Drug pharmacokinetics have improved and since 2006, single daily pill formulations with fewer drug interactions have been available.^2^ Mortality soon after starting ART is strongly influenced by the proportion of patients who start ART with severe immunodeficiency (late presentation), but reductions in the proportion of such patients starting ART do not explain our findings, because we controlled for previous AIDS and for CD4 cell count at ART initiation. Better management of patients with late presentation of HIV infection and more general improvements in health care for people living with HIV could have contributed to improved survival. With the perception that HIV-positive people will live into old age, clinicians are screening for and treating comorbidities more aggressively, including common disorders such as cardiovascular disease, hepatitis C, and cancer. Increasing life expectancy might encourage patients to engage in risk reduction programmes, to cease smoking, and to increase adherence to ART.^15^ In the USA, improved survival after 2010 could in part be a result of the introduction of the National HIV/AIDS Strategy, which aimed to increase access to care, improve health outcomes, and address health inequities among people living with HIV.

Rates of AIDS deaths during the first year of ART were substantially lower in the most recent period, which is probably caused by declining rates of more serious AIDS events, such as AIDS-defining malignancies.^6^ As we reported previously,^16^ the proportion of deaths from AIDS has declined over time. The substantial decline in cardiovascular mortality could be a result of more aggressive screening and treatment of cardiovascular risk factors, decreasing contraindications for lipid-lowering medications, especially statins (because of drug interactions or poor overall condition), and reduced use of abacavir in individuals who have high viral loads, are HLA B5701 positive or are at high risk of myocardial infarction.^17^ Moreover, the incidence of cardiovascular disease has decreased in the general population over time, and therapeutic interventions have improved.^18^

Between 1996–99 and 2008–10, life expectancy in people living with HIV starting ART increased by around 10 years for both sexes, in Europe and North America. However, the 12-year improvements that we found were less than the 15–24 year increases over similar time periods reported by other studies in the UK and North America.^19^, ^20^, ^21^, ^22^ Two of these studies examined trends by period of follow-up^21^, ^22^ and two^19^, ^20^ did not control for duration of ART, which tends to increase estimated life expectancy in earlier periods relative to later periods^23^ because mortality is higher soon after the start of ART than after successful treatment for a number of years. Estimates of life expectancy in patients who survive the first year of ART are much higher than at ART initiation, reflecting the importance of starting therapy early in the course of HIV infection.^20^, ^23^

We analysed data for many people living with HIV who were receiving routine clinical care in western Europe, the USA, and Canada. Some cohorts, such as the Danish HIV cohort, FHDH (France), and ATHENA (Netherlands) cover most of their countries. Others are regional but are representative of public care for the areas in which they operate. Therefore, our findings should be generalisable to treated people living with HIV in high-income settings. We compared patients with the same potential years of follow-up between calendar periods and accounted for heterogeneity in death rates between cohorts.^24^ Our results might be affected by confounding: patient characteristics have changed during the 20 years that ART has been available, with a smaller proportion of infections in people who inject drugs in more recent years and changing patterns of migration from sub-Saharan Africa. Transmission group is sometimes misclassified, and transmission for people who inject drugs does not necessarily imply continuing drug use. Outcomes in patients lost to follow-up within 3 years of starting ART are uncertain, but most cohorts link to death registries. CD4 cell count and viral load 1 year after starting ART were missing for some patients who might have been less engaged in care and likely to have worse prognosis. Mortality was estimated on pooled data and therefore the estimated life expectancy reflects patients' average experience. Estimates of life expectancy are sensitive to mortality in the oldest age groups, for which data are sparse. Our study only includes patients who started ART, whereas most deaths in people with HIV infection occur in the untreated population.

Our study tracks the progress made in treating people living with HIV between 1996 and 2013. Monitoring survival can clarify when and how improvements were achieved, and provides a benchmark against which current or future interventions, such as treatment with integrase inhibitors, guidelines recommending earlier treatment, or limiting CD4 cell count and viral load monitoring in stable patients, can be measured. Prognostic information is important to patients, their relatives, and clinicians, and can be used to inform health-care planning at the individual, clinic, and government levels. Improvements in survival with better ART regimens could provide evidence to policy makers that modern palatable and effective treatments should continue to be used in preference to older antiretroviral drugs that are becoming available as cheaper generics. Information about life expectancy in people living with HIV and the knowledge that it could be approaching that of the general population is important to motivate at-risk individuals to test for HIV and convince those infected to start ART immediately, and might decrease stigmatisation of people living with HIV and help them to obtain insurance or employment.

Since modern ART is highly effective and has low toxicity, the excess mortality in people living with HIV is unlikely to be addressed by further development of antiretroviral drugs. Instead, lifestyle issues that affect adherence to ART and non-AIDS mortality, and diagnosis and treatment of comorbidities in people living with HIV will need to be addressed. Interventions are needed to promote modern therapy to vulnerable populations, such as people who inject drugs, who currently do not fully benefit from ART. Improved access to opioid substitution treatment programmes and direct acting antiviral drugs for hepatitis C virus co-infection should be a priority for this group.^25^

Continued efforts are required to address late diagnosis and presentation to care to decrease mortality soon after starting ART, and support lifelong adherence to ART. Treatment guidelines changed in 2015 after results of the START trial showed clear benefits of immediate versus deferred treatment.^26^ Most future patients diagnosed with HIV are likely to start ART immediately (both for their own health and to prevent transmission to others), but this will only result in improved survival if the problems of late HIV diagnosis and access to care are addressed.

Correspondence to: Mr Adam Trickey, School of Social and Community Medicine, University of Bristol, Bristol BS8 2PS, UK **adam.trickey@bristol.ac.uk**

## References

[1] Camacho R, Teofilo E (2011). Antiretroviral therapy in treatment-naive patients with HIV infection. Curr Opin HIV AIDS.

[2] Astuti N, Maggiolo F (2014). Single-tablet regimens in HIV therapy. Infect Dis Ther.

[3] Benson CA, Kaplan JE, Masur H (2004). Treating opportunistic infections among HIV-infected adults and adolescents: recommendations from CDC, the National Institutes of Health, and the HIV Medicine Association/Infectious Diseases Society of America. MMWR Recomm Rep.

[4] May MT, Sterne JA, Costagliola D (2006). HIV treatment response and prognosis in Europe and North America in the first decade of highly active antiretroviral therapy: a collaborative analysis. Lancet.

[5] CASCADE Collaboration (2003). Determinants of survival following HIV-1 seroconversion after the introduction of HAART. Lancet.

[6] Monforte A, Abrams D, Pradier C (2008). HIV-induced immunodeficiency and mortality from AIDS-defining and non-AIDS-defining malignancies. AIDS.

[7] Harrison KM, Song R, Zhang X (2010). Life expectancy after HIV diagnosis based on national HIV surveillance data from 25 states, United States. J Acquir Immune Defic Syndr.

[8] McManus H, O'Connor CC, Boyd M (2012). Long-term survival in HIV positive patients with up to 15 years of antiretroviral therapy. PLoS One.

[9] Smith CJ, Ryom L, Weber R (2014). Trends in underlying causes of death in people with HIV from 1999 to 2011 (D:A:D): a multicohort collaboration. Lancet.

[10] May MT, Ingle SM, Costagliola D (2014). Cohort profile: Antiretroviral Therapy Cohort Collaboration (ART-CC). Int J Epidemiol.

[11] Ingle SM, May MT, Gill MJ (2014). Impact of risk factors for specific causes of death in the first and subsequent years of antiretroviral therapy among HIV-infected patients. Clin Infect Dis.

[12] Abgrall S, Ingle SM, May MT (2013). Durability of first ART regimen and risk factors for modification, interruption or death in HIV-positive patients starting ART in Europe and North America 2002–2009. AIDS.

[13] Gill VS, Lima VD, Zhang W (2010). Improved virological outcomes in British Columbia concomitant with decreasing incidence of HIV type 1 drug resistance detection. Clin Infect Dis.

[14] Hughes CA, Robinson L, Tseng A, MacArthur RD (2009). New antiretroviral drugs: a review of the efficacy, safety, pharmacokinetics, and resistance profile of tipranavir, darunavir, etravirine, rilpivirine, maraviroc, and raltegravir. Expert Opin Pharmacother.

[15] Rockstroh JK, Gatell J, Landman R, Antinori A (2010). Management of late-presenting patients with HIV infection. Antivir Ther.

[16] Vandenhende MA, Roussillon C, Henard S (2015). Cancer-related causes of death among HIV-infected patients in France in 2010: evolution since 2000. PLoS One.

[17] Sabin CA, Worm SW, D:A:D Study Group (2008). Use of nucleoside reverse transcriptase inhibitors and risk of myocardial infarction in HIV-infected patients enrolled in the D:A:D study: a multi-cohort collaboration. Lancet.

[18] O'Flaherty M, Buchan I, Capewell S (2013). Contributions of treatment and lifestyle to declining CVD mortality: why have CVD mortality rates declined so much since the 1960s?. Heart.

[19] Patterson S, Cescon A, Samji H (2015). Life expectancy of HIV-positive individuals on combination antiretroviral therapy in Canada. BMC Infect Dis.

[20] May M, Gompels M, Delpech V (2011). Impact of late diagnosis and treatment on life expectancy in people with HIV-1: UK Collaborative HIV Cohort (UK CHIC) Study. BMJ.

[21] Samji H, Cescon A, Hogg RS (2013). Closing the gap: increases in life expectancy among treated HIV-positive individuals in the United States and Canada. PLoS One.

[22] Marcus JL, Chao CR, Leyden WA (2016). Narrowing the gap in life expectancy between HIV-infected and HIV-uninfected individuals with access to care. J Acquir Immune Defic Syndr.

[23] Johnson LF, Mossong J, Dorrington RE (2013). Life expectancies of South African adults starting antiretroviral treatment: collaborative analysis of cohort studies. PLoS Med.

[24] May MT, Hogg RS, Justice AC (2012). Heterogeneity in outcomes of treated HIV-positive patients in Europe and North America: relation with patient and cohort characteristics. Int J Epidemiol.

[25] Buse K, Albers E, Phurailatpam S (2016). HIV and drugs: a common, common-sense agenda for 2016. Lancet Glob Health.

[26] Lundgren JD, Babiker AG, Insight Start Study Group (2015). Initiation of antiretroviral therapy in early asymptomatic HIV infection. N Engl J Med.

