# Supplementary material for: Survival of HIV-positive patients starting antiretroviral therapy between 1996 and 2013: a collaborative analysis of cohort studies
Source: Lancet HIV. 2017 May 10;4(8):e349–56. doi: 10.1016/S2352-3018(17)30066-8 (PMC5555438; doi:10.1016/S2352-3018(17)30066-8)
Supplement: Supplementary appendix [file mmc1.pdf]

THE LANCET
HIV

Supplementary appendix

This appendix formed part of the original submission and has been peer reviewed. We post it as supplied by the authors.

Supplement to: The Antiretroviral Therapy Cohort Collaboration. Survival of HIV-positive patients starting antiretroviral therapy between 1996 and 2013: a collaborative analysis of cohort studies. *Lancet HIV* 2017; published online May 10. http://dx.doi.org/10.1016/S2352-3018(17)30066-8.

Web Appendix

Webtable 1: Adjusted* all-cause mortality hazard ratios by period of starting ART (vs. 2000-03) in subgroups of patients defined by their characteristics at the time of starting ART: (i) first year of ART (upper panel) (ii) 2nd and 3rd years after starting ART (lower panel).

		Period of starting ART				Per period
		1996-99	2000-3	2004-7	2008-10	
**1st year of follow-up**	**N**					
**All**	**88,504**	**1.02 (0.91, 1.14)**	**1**	**0.97 (0.87, 1.09)**	**0.71 (0.61, 0.83)**	**0.90 (0.87, 0.95)**
Age 16-29 (years)	15,403	1.19 (0.78, 1.83)	1	0.73 (0.44, 1.22)	1.10 (0.60, 2.01)	0.89 (0.74, 1.08)
Age 30-39	34,993	1.12 (0.92, 1.37)	1	1.05 (0.83, 1.32)	0.63 (0.44, 0.91)	0.89 (0.82, 0.98)
Age 40-49	24,048	0.89 (0.74, 1.10)	1	0.91 (0.75, 1.10)	0.69 (0.53, 0.89)	0.94 (0.87, 1.01)
Age 50-59	10,190	1.00 (0.79, 1.27)	1	0.90 (0.71, 1.13)	0.62 (0.46, 0.84)	0.87 (0.80, 0.96)
Age ≥ 60	3,870	0.96 (0.69, 1.35)	1	1.05 (0.77, 1.42)	0.71 (0.49, 1.03)	0.93 (0.83, 1.05)
CD4 < 100 cells/mL	21,378	1.00 (0.86, 1.15)	1	1.05 (0.90, 1.22)	0.82 (0.66, 1.01)	0.97 (0.91, 1.03)
CD4 100-199 cells/mL	17,315	1.00 (0.78, 1.27)	1	0.92 (0.72, 1.16)	0.49 (0.33, 0.71)	0.86 (0.78, 0.94)
CD4 200-349 cells/mL	28,379	1.09 (0.81, 1.47)	1	0.82 (0.61, 1.09)	0.60 (0.42, 0.85)	0.83 (0.74, 0.92)
CD4 ≥ 350 cells/mL	21,432	1.12 (0.75, 1.68)	1	1.07 (0.67, 1.69)	1.09 (0.69, 1.71)	0.99 (0.86, 1.14)
N. America	16,349	0.99 (0.82, 1.18)	1	0.96 (0.80, 1.16)	0.62 (0.49, 0.79)	0.89 (0.83, 0.96)
Europe	72,155	1.02 (0.88, 1.17)	1	0.95 (0.83, 1.09)	0.75 (0.62, 0.91)	0.93 (0.88, 0.98)
Male PWID	8,772	1.00 (0.73, 1.38)	1	1.25 (0.89, 1.77)	0.89 (0.51, 1.55)	1.04 (0.90, 1.20)
Female PWID	2,555	0.94 (0.43, 2.06)	1	1.16 (0.52, 2.56)	1.93 (0.22, 1.53)	1.23 (0.90, 1.69)
MSM	25,217	0.83 (0.63, 1.08)	1	0.89 (0.69, 1.17)	0.69 (0.49, 0.97)	0.96 (0.87, 1.06)
Heterosexual Male	14,102	0.87 (0.65, 1.16)	1	1.00 (0.77, 1.30)	0.60 (0.40, 0.91)	0.95 (0.85, 1.06)
Heterosexual Female	15,591	1.78 (1.21, 2.64)	1	1.22 (0.82, 1.81)	1.10 (0.65, 1.87)	0.89 (0.51, 1.54)
**2nd/3rd years of follow-up**						
**All**	**81,608**	**1.21 (1.10, 1.45)**	**1**	**0.79 (0.71, 0.89)**	**0.57 (0.49, 0.67)**	**0.78 (0.75, 0.82)**
Age 16-29 (years)	14,319	1.41 (0.98, 2.03)	1	0.74 (0.47, 1.16)	0.44 (0.21, 0.93)	0.70 (0.59, 0.84)
Age 30-39	32,390	1.15 (0.96, 1.37)	1	0.77 (0.61, 0.96)	0.48 (0.34, 0.68)	0.79 (0.72, 0.86)
Age 40-49	22,143	1.26 (1.06, 1.50)	1	0.70 (0.57, 0.86)	0.56 (0.43, 0.74)	0.76 (0.70, 0.82)
Age 50-59	9,337	1.34 (1.06, 1.68)	1	0.99 (0.78, 1.25)	0.65 (0.47, 0.88)	0.82 (0.75, 0.89)
Age ≥ 60	3,419	0.92 (0.65, 1.29)	1	0.77 (0.55, 1.06)	0.54 (0.37, 0.78)	0.84 (0.74, 0.94)
CD4 < 100 cells/mL	19,085	1.18 (1.02, 1.37)	1	0.77 (0.64, 0.91)	0.63 (0.49, 0.81)	0.81 (0.76, 0.87)
CD4 100-199 cells/mL	15,966	1.18 (0.96, 1.46)	1	0.63 (0.50, 0.80)	0.41 (0.28, 0.59)	0.72 (0.65, 0.79)
CD4 200-349 cells/mL	26,577	1.51 (1.19, 1.92)	1	1.07 (0.84, 1.36)	0.72 (0.53, 0.97)	0.81 (0.74, 0.89)
CD4 ≥ 350 cells/mL	19,980	1.03 (0.77, 1.36)	1	0.87 (0.62, 1.22)	0.48 (0.32, 0.74)	0.82 (0.73, 0.92)
N. America	15,306	1.31 (1.12, 1.53)	1	0.70 (0.58, 0.85)	0.62 (0.50, 0.78)	0.76 (0.71, 0.82)
Europe	66,302	1.13 (0.99, 1.29)	1	0.83 (0.72, 0.96)	0.51 (0.41, 0.64)	0.81 (0.77, 0.86)
Male PWID	7,813	0.86 (0.67, 1.11)	1	0.74 (0.54, 1.00)	0.54 (0.33, 0.90)	0.90 (0.80, 1.02)
Female PWID	2,290	0.65 (0.40, 1.06)	1	0.74 (0.43, 1.25)	0.37 (0.13, 1.05)	0.95 (0.76, 1.20)
MSM	23,718	1.19 (0.90, 1.55)	1	0.78 (0.58, 1.05)	0.54 (0.37, 0.81)	0.79 (0.71, 0.88)
Heterosexual Male	12,862	1.76 (1.31, 2.35)	1	1.12 (0.82, 1.52)	0.61 (0.37, 1.00)	0.76 (0.67, 0.86)
Heterosexual Female	14,447	1.27 (0.85, 1.91)	1	1.07 (0.72, 1.57)	0.73 (0.40, 1.32)	0.88 (0.74, 1.04)

*All models mutually adjusted for sex, age, PWID, AIDS status, CD4 and viral load as appropriate and stratified by cohort.

Webtable 2: Adjusted* hazard ratios (95% CI) with numbers of events for (i) regimen failure – defined as switching regimen within 6 months of a viral load measurement >1000 copies/mL (ii) death following regimen failure

	Period of starting ART				Per period
	1996-99	2000-03	2004-07	2008-10	
Regimen failure	N: 3,852 (16%)	N: 3,369 (13%)	N: 2,079 (9%)	N: 792 (6%)	
	1.23 (1.18, 1.29)	1	0.69 (0.65, 0.73)	0.45 (0.42, 0.49)	0.73 (0.72, 0.75)
Death (after regimen failure)	N: 143 (4%)	N: 146 (4%)	N: 70 (3%)	N: 29 (4%)	
	1.00 (0.79, 1.27)	1	0.86 (0.65, 1.15)	1.07 (0.71, 1.60)	0.98 (0.87, 1.09)

*mutually adjusted for age, sex, CD4, viral load, AIDS and PWID transmission risk, and stratified by cohort.

Webtable 3: Expected age at death for PLWH starting ART aged 20 years (years) (95% CI), for men and women by calendar period of starting ART, estimated at exact age 20 years old, for (i) all cohorts; (ii) North American cohorts; and (iii) European cohorts.

	Period of starting ART				Per period
	1996-99	2000-03	2004-07	2008-10	increase
**Based on mortality rates during the 1st 3 years of ART**					
**All patients**					
Men	54.4 (53.0, 55.7)	55.0 (53.6, 56.3)	58.8 (57.6, 60.1)	64.0 (63.1, 65.0)	3.3 (0.2, 6.5)
Women	58.0 (56.8, 59.3)	60.8 (59.7, 62.0)	62.3 (61.2, 63.4)	66.6 (65.8, 67.4)	2.8 (1.1, 4.4)
**Patients in North America**					
Men	47.6 (46.1, 49.1)	48.7 (47.2, 50.1)	52.6 (51.2, 54.0)	57.2 (56.0, 58.4)	3.3 (0.8, 5.8)
Women	46.2 (44.5, 48.0)	47.6 (45.9, 49.3)	48.1 (46.4, 49.8)	58.1 (56.9, 69.4)	3.6 (-3.1, 10.4)
**Patients in Europe**					
Men	58.1 (56.8, 59.4)	58.4 (57.1, 59.8)	61.6 (60.3, 62.8)	67.6 (66.7, 68.5)	3.2 (-0.7, 7.1)
Women	59.4 (38.2, 60.7)	62.4 (61.4, 63.5)	64.4 (63.4, 65.4)	67.9 (67.2, 68.7)	2.8 (1.8, 3.8)
**Based on mortality rates during the 2nd and 3rd years of ART**					
**All patients**					
Men	62.5 (61.3, 63.6)	64.5 (63.4, 65.6)	69.5 (68.6, 70.4)	73.1 (72.5, 73.8)	3.7 (2.2, 5.3)
Women	67.1 (66.0, 68.2)	69.7 (68.7, 70.6)	71.5 (70.6, 72.4)	76.0 (75.5, 76.5)	2.9 (1.2, 4.6)
**Patients in North America**					
Men	56.1 (54.9, 57.4)	58.6 (57.3, 59.8)	64.0 (63.0, 65.0)	65.9 (65.0, 66.8)	3.5 (1.4, 5.6)
Women	51.5 (49.8, 53.3)	57.5 (56.0, 58.9)	55.4 (53.8, 57.0)	63.2 (62.2, 64.3)	3.3 (-2.3, 8.9)
**Patients in Europe**					
Men	67.8 (66.6, 68.9)	69.6 (68.6, 70.7)	73.7 (72.8, 74.6)	79.0 (78.4, 79.5)	3.8 (1.5, 6.1)
Women	69.4 (68.4, 70.4)	71.4 (70.5, 72.3)	74.0 (73.2, 74.8)	78.3 (77.9, 78.8)	3.0 (1.4, 4.6)
